# Diagnostic dilemma of pulmonary tuberculosis among adults with severe mental illness in Beijing, China

**DOI:** 10.1186/s12879-017-2190-6

**Published:** 2017-01-18

**Authors:** Li Wang, Zhiguo Zhang, Qiuli Yan, Jie Lu, Baoyin Gao, Yanlin Zhao, Yu Pang

**Affiliations:** 1 Changping Tuberculosis Dispensary, Beijing, People’s Republic of China; 2 Changping Hospital of Integrated Chinese and Western Medicine, Beijing, People’s Republic of China; 3 Beijing Pediatric Research Institute, Beijing Children’s Hospital, Capital Medical University, Beijing, People’s Republic of China; 4 Beijing Chest Hospital, Capital Medical University, Beijing, People’s Republic of China; 5 National Center for Tuberculosis Control and Prevention, Chinese Center for Disease Control and Prevention, Beijing, People’s Republic of China

## Abstract

**Background:**

Although the prevalence of tuberculosis has decreased significantly over the past decades, the certain populations with mental illness are at increased risk for tuberculosis infection and transmission. However, no studies have examined the performance of different laboratory examination methods among people with severe mental illness in China.

**Methods:**

In this study, we firstly performed a retrospective study to evaluate the feasibility of three routine laboratory methods, including sputum microscopy, solid culture and GeneXpert, to diagnose tuberculosis patients with mental illness.

**Results:**

During August 2010 and March 2013, a total of 251 TB patients based on clinical and radiographic criteria with severe mental illness were enrolled in this study. The majority of patients was homeless (97/251, 38.6%), and the other 62 (24.7%) and 92 (36.7%) were from urban and rural region, respectively. The most frequently diagnosed mental illness was schizophrenia, accounting for 84.1% (211/251) of patients available for analysis. In addition, the laboratory received 753 sputum samples collected from these 251 TB patients, of which 76.0% (572/753) of samples were classified as salivary sputum, which were unqualified for microscopy and culture. When the test results were analyzed by patients, the positive numbers of TB patients detected by sputum microscopy, solid culture and GeneXpert were 3 (1.2%), 5 (2.0%) and 5 (2.0%), respectively.

**Conclusions:**

In conclusion, our findings reveal that the current laboratory examinations based on sputum samples seem not to be suitable for the diagnosis of active TB in the persons with severe mental illness. The products using a non-invasive specimen such as urine deserve further evaluation, which may generate benefit for the early diagnosis of TB in this special population.

## Background

Tuberculosis, caused by *Mycobacterium tuberculosis* complex (MTBC), is still a major public health problem worldwide [1, 2]. It is estimated that 9.6 million new tuberculosis cases emerged in 2014, and 1.5 million patients died from TB [1]. Similar to tuberculosis, mental disorders have also become a global challenge [3–5]. The relationship between tuberculosis and severe mental illness is complex, while the vulnerability factors in people with severe mental illness increase the risk of tuberculosis, including homelessness, poverty, and alcohol/substance abuse [6–9]. Hence, it is reasonable to hypothesize that tuberculosis is a relatively common condition among patients with severe mental disorder [6]. The previous reported TB outbreaks in these patients have been challenging the current TB control program [7], which has paid less attention to this high TB risk group.

China has the third largest number of tuberculosis patients worldwide, with an estimated incidence of 68 per 100,000 populations in 2014 [1]. Although the prevalence of tuberculosis has decreased significantly over the past decades, the certain populations with mental illness are at increased risk for tuberculosis infection and transmission [10–12]. A number of studies have shown that people with severe mental illness have higher than expected rates of tuberculosis compare with the general population, which serves as an important contributor to the higher mortality [13]. Nevertheless, the management of tuberculosis patients with severe mental illness has been a low priority, and no systematic diagnostic and treatment flow for tuberculosis has been established for this special population in China. Considering people with severe mental disorders may have obvious difficulties in communication and cognitive functioning, the most obvious difficulty for clinicians is how to diagnose the tuberculosis with limited laboratory test results and clinical features. Unfortunately, no studies have examined the performance of different laboratory examination methods among people with severe mental illness in China.

In this study, we performed a retrospective study to evaluate the feasibility of three routine laboratory methods, including sputum microscopy, solid culture and GeneXpert, to diagnose tuberculosis patients with mental illness for the first time. Our aim was to provide evidence for generating appropriate diagnostic scheme for people with mental illness.

## Methods

### Study participants

We conducted a retrospective study at Changping Hospital of Integrated Chinese and Western Medicine in China on clinically diagnosed TB patients with severe mental illness between August 2010 and March 2013. This hospital was the only public sector psychiatric hospital providing health care service of the clinical diagnosis and treatment of tuberculosis in Beijing. All the TB suspects with severe mental illness were transferred to this hospital for further diagnosis of TB. The diagnosis of patients with mental illness followed the Chinese Classification of Mental Disorders Version 3 (CCMD-3), Diagnostic and Statistical Manual of Mental Disorders (DSM) and International Classification of Diseases (ISD) [14, 15], and the individuals with severe mental illness included six different types: schizophrenia, bipolar disorder, schizoaffective disorder, affective disorder, paranoid disorders and intellectual disability.

**Fig. 1:**
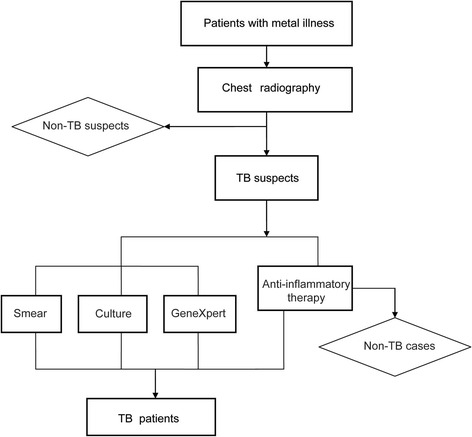
Diagnosis flow of TB patients with mental illness

All the patients with severe mental illness were firstly scanned with computed tomography (CT), and those with radiological tuberculosis features were referred to Changping Hospital of Integrated Chinese and Western Medicine for further examinations as in-patients. Three sputum samples were collected in the morning, evening and on-the-spot from TB suspects, respectively. The sputum samples were then transported to Changping Tuberculosis Dispensary for laboratory examinations, including staining of TB smears for acid-fast bacilli (AFB), conventional cultures for *M. tuberculosis*, and GeneXpert (Fig. 1). The definitions of confirmed TB cases and clinically diagnosed TB cases followed the National Guidelines on Diagnosis of Tuberculosis in China. The confirmed TB cases included the TB suspect with positive smear and/or culture results, while the clinically diagnosed TB cases were diagnosed on the basis of clinical and radiological indicators [16]. The demographic information was obtained by reviewing the medical record.

### Laboratory examination

Fluorescent smear microscopy was performed directly on all samples as described previously [17]. In addition, two of three sputum samples from each patient, which had high positive grades of smear microscopy, and good quality, were selected for further solid culture. The sputum samples were homogenized and digested in N-acetyl-L-cysteine-NaOH-Na citrate (2.0% final concentration), and vortexed for 30 s. After incubation for 15 min at room temperature, the prepared sputum samples were neutralized with 45 mL of phosphate buffer (PBS, 0.067 mol/L, pH = 7.4), with subsequent centrifugation at 3000 g for 15 min. The supernatants were discarded and the precipitates were resuspended in 1 mL PBS. 0.1 to 0.15 ml of this suspension was inoculated onto modified Lowenstein-Jensen (L-J) medium according to the previous report [18]. All culture media were incubated at 37 °C in an atmosphere of 5 to 10% CO_2_. All solid slants were tested either until found to be positive or for 8 weeks.

The clinical samples were also detected by GeneXpert, an automatic molecular test for MTB by amplifying an MTB specific sequence. For testing by the GeneXpert, the residual of each concentrated sputum sample was mixed with 2.0 mL GeneXpert MTB/RIF sample reagent, and incubated at room temperature for 15 min. 2.0 mL of the mixed sample was then transferred to a test cartridge and loaded onto the GeneXpert instrument. Results for the presence of *M. tuberculosis* were automatic generated by the instrument in approximately 120 min.

## Results

### Patients enrollment

A total of 251 TB patients with severe mental illness took part in this study, including 174 men (69.3%) and 77 women (30.7%). Subjects were between the ages of 19 and 79 years, with a median age of 43 years. The mean body mass index (BMI) analysis showed that 3.6% of patients had BMI values lower than 18.5, indicating wasting. The majority of patients was homeless (97/251, 38.6%), and the other 62 (24.7%) and 92 (36.7%) were from urban and rural region, respectively. In addition, the most frequently diagnosed mental illness was schizophrenia, accounting for 84.1% (211/251) of patients available for analysis; 4.8% (12/251) and 11.2% (28/251) of patients had bipolar disorder and other psychiatric diagnosis, respectively (Table 1).

**Table 1:** Characteristics of tuberculosis patients with severe mental illness enrolled in this study

Characteristics	No. (%)
Sex	
Male	174 (69.3)
Female	77 (30.7)
Age group (years)	
< 25	23 (9.2)
25–44	114 (45.4)
45–64	95 (37.8)
≥ 65	19 (7.6)
Residence	
Urban	62 (24.7)
Rural	92 (36.7)
Homeless	97 (38.6)
BMI (kg/m^2^)^a^	
< 18.5	9 (3.6)
18.5–23.9	149 (59.4)
24.0–27.9	78 (31.1)
≥ 28.0	6 (2.4)
Psychiatric diagnosis	
Schizophrenia	211 (84.1)
Bipolar disorder	12 (4.8)
Others^b^	28 (11.2)

^a^ *BMI* Body Mass Index

^b^Others include schizoaffective disorder, affective disorder, paranoid disorders and mental retardation

### Quality of sputum samples

**Fig. 2:**
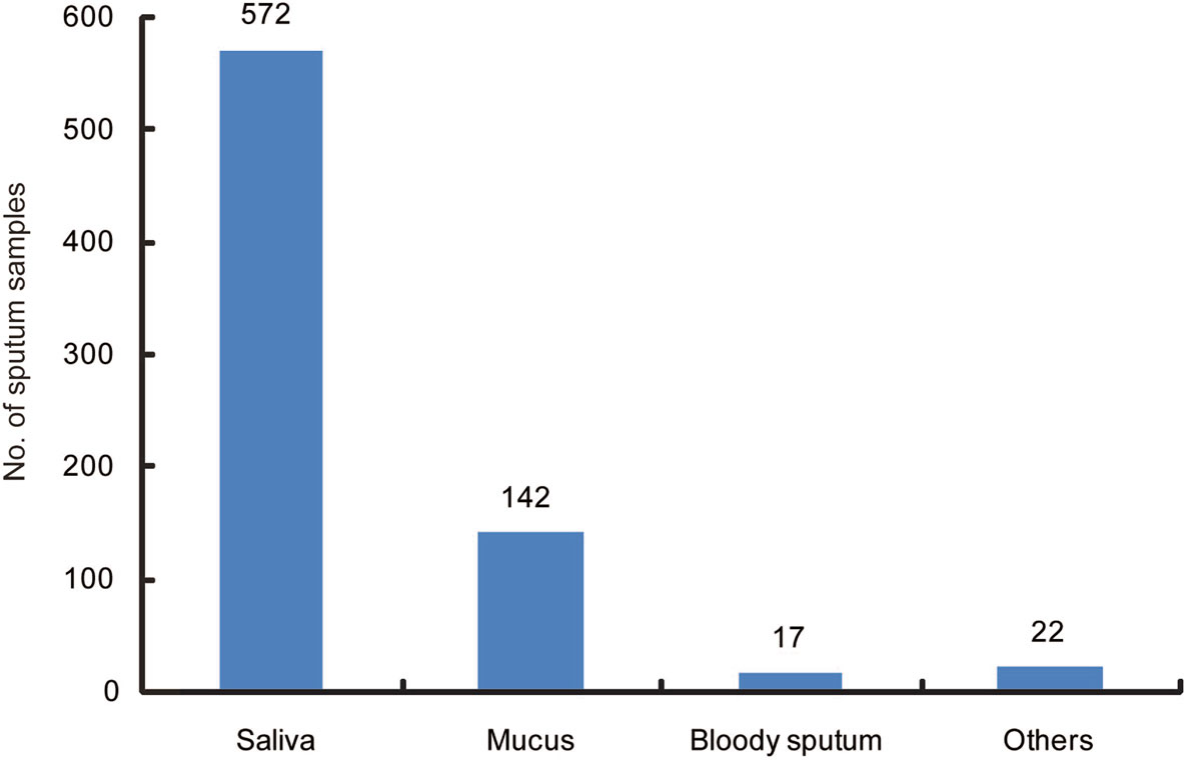
Classification of sputum samples collected from TB patients with mental illness

During August 2010 and March 2013, the laboratory received 753 sputum samples collected from 251 diagnosed TB patients. We further assessed the quality of sputum samples. According to the National guidelines for TB laboratories from China Center for Disease Control and Prevention, the sputum samples were divided into salivary sputum, mucus sputum, bloody sputum and caseous sputum. As shown in Fig. 2, 76.0% (572/753) samples used for bacterial examination were salivary sputum. Only 142 (18.9%) out of 753 sputa were classified as mucus sputum, which were qualified for microscopy and culture. In addition, we also observed 22 (2.9%) transparent samples with low viscosity, which were possibly water or urine. All these samples were submitted to sputum microscopy, mycobacteria culture and GeneXpert as previous described.

### Results of laboratory examinations

As summarized in Table 2, the laboratory performed 753 sputum smears, 502 solid cultures and 502 GeneXpert tests, respectively. Six out of 753 smears were positive, demonstrating a positive rate of 0.8%. Solid culture identified eight positive results (1.6%) from 502 cultures. The GeneXpert assay exhibited the best positive rate (9/502, 1.8%), which yielded one more positive sample than solid culture. When the test results were analyzed by patients, the positive numbers of TB patients detected by sputum microscopy, solid culture and GeneXpert were 3 (1.2%), 5 (2.0%) and 5 (2.0%), respectively.

**Table 2:** Results of laboratory tests among the TB patients with severe mental illness

Clinical test	Results per sputum sample (%)			Results per patient (%)		
	Completed	Positive	Negative	Completed	Positive	Negative
Sputum microscopy	753	6 (0.8)	747 (99.2)	251	3 (1.2)	248 (98.8)
Solid culture	502	8 (1.6)	494 (98.4)^a^	251	5 (2.0)	246 (98.0)
GeneXpert	502	9 (1.8)	493 (98.2)	251	5 (2.0)	246 (98.0)

^a^The negative solid culture results include culture-negative and contaminated results

## Discussion

**Fig. 3:**
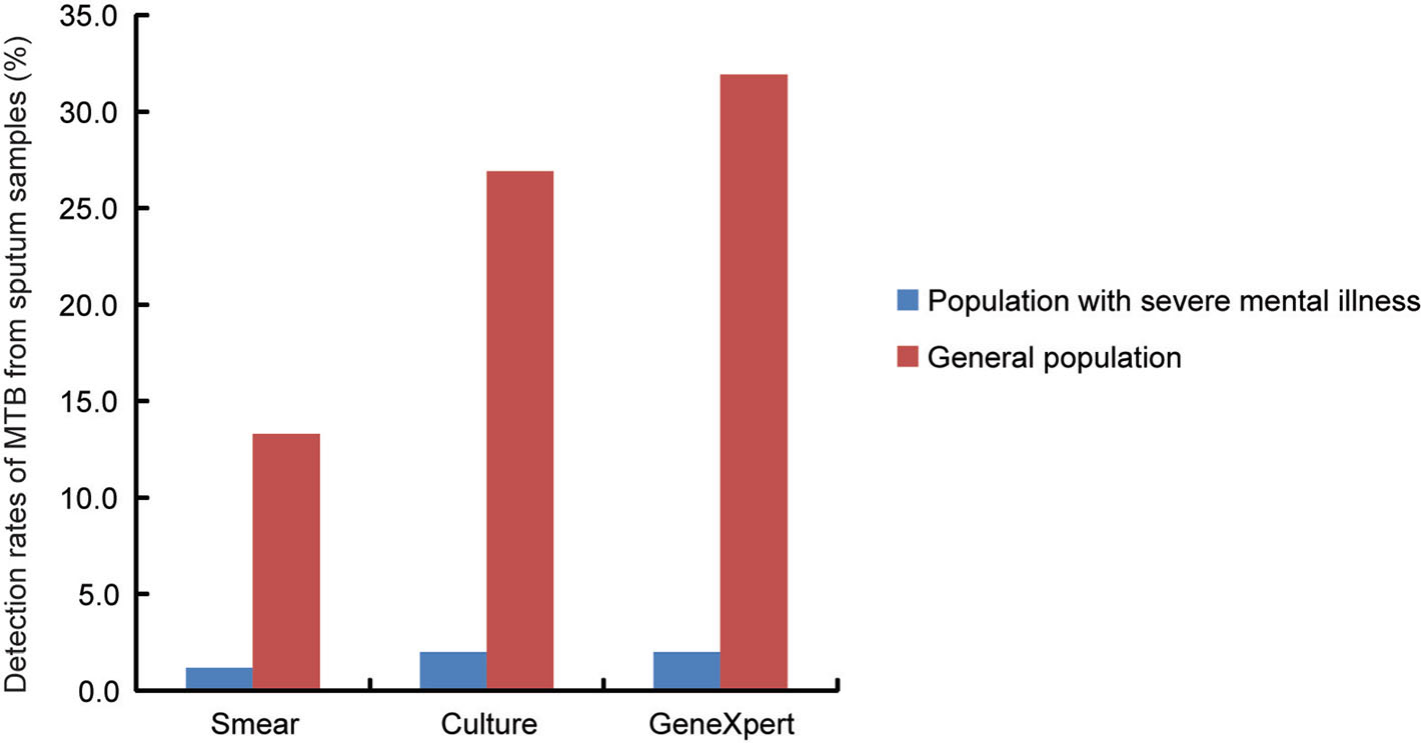
Comparison of detection rates of MTB from sputum samples by smear, culture and GeneXpert between population with severe mental illness and general population

In recent years, China meets the public health challenge from both tuberculosis and health illness [19, 20]. Previous literatures have demonstrated that rate of tuberculosis are higher in individuals with medical illness than in the general populations [6, 10]. Early diagnosis of TB cases among this high risk group will prevent potential sources of infection at the earliest possible time. Unfortunately, our data demonstrated that no more than 2.0% of tuberculosis cases with severe mental illness could be identified by laboratory examination, which was significantly lower than that among the general population (13.3% for smear, 26.9% for culture, and 31.9% for GeneXpert) in China (Fig. 3) [21]. There were several potential explanations for this finding. First, the rate of smear positive results is always lower in salivary specimens when compared with that in mucous ones [22]. However, three quarters of specimens collected in this study were qualified as “saliva”. In addition, we also found that a small proportion (2.9%) of inappropriate specimens may be water or urine rather than sputum. Taken together, the high percentage of unqualified samples collected from TB patients with severe mental illness may be the major reason for the low detection rate of bacteriologically confirmed TB cases. Second, the policy of regular radiographic screening in the patients with mental illness was active and enhanced case finding strategy, and the TB patients could be diagnosed in the early active stage, which may be another factor contributed to the low proportion of patients with bacteriological evidence.

Because the signs and symptoms of TB are shared with many other diseases, TB misdiagnosis is frequently observed in the clinical diagnosed TB cases [23]. Considering the diagnosis TB in mental disorders is predominantly based on chest radiography combined with clinical symptoms, it is essential to provide more reliable laboratory results for confirmation of the TB patients in this special population. On one hand, collection of qualified sputum samples from TB suspects were a major issue that should be taken into account. Unfortunately, persons with severe mental illness often exhibit poor social and cognitive functioning; it is possible that they are more likely to swallow sputum rather than expectorate it. Although the sputum induction, a technique for collecting sputum samples from lower airways by inhaling an aerosol of saline over different timeperiods, has been a useful alternative for improving the quality of sputum [24], it is rarely used in patients with severe mental illness due to unexpected risk of medical accidents. Hence, the question becomes how to develop and utilize the new laboratory examinations based on other clinical specimens rather than sputum samples. When compared with sputum, urine is an ideal specimen type as it is non-invasive and easy to collect [25]. In addition, previous literatures have demonstrated that this matrix contains biomarkers and metabolites from systemic infectious diseases [26]. Recently, several commercial TB diagnostic kits are available to detect the presence of lipoarabinomannan (LAM) in urine, which is proposed as a promising biomarker for early detection of TB [25–27]. Preliminary evaluation results have demonstrated that these products have high specificity and moderate sensitivity detect TB in adults [26]. These urine-based assays therefore provide an available solution for informing on active TB disease among patients with severe mental illness.

The homeless population is usually considered a group at high risk for TB infection [28]. Consistent to previous results, we observed that nearly 40% of TB cases were from homeless population with severe mental illness in this study. Given the relatively low proportion of homeless population in China, our data indicate that the homeless may be an important risk factor for TB in this special population. Due to the exposed constellation of risk factors and lack of seeking medical services, we hypothesized that TB in the homeless population with severe mental illness will represent an increasing public health concern. Hence, there is an urgent need to improve access to mental health care and housing for people with severe mental illness so that they less likely to become homeless and be vulnerable to developing TB. In addition, regular screening this population for TB may play an essential role in preventing TB transmission and morbidity.

We also realized several obvious limitations in this study. First, our results are limited by the small size of the sample. Because this study was performed in Beijing, a metropolis with low TB prevalence, there were less than 4000 new tuberculosis cases emerging in 2013 in the general population, which is associated with the small number of TB case herein. Second, the definition of tuberculosis case is majorly on the basis of the chest radiography and clinical symptoms. Therefore some patients misdiagnosed as TB may be enrolled in this research, which may disturb the analysis results. Third, pervious evidence reveals that about half of individuals with severe mental illnesses will develop a substance use disorder during their lives [29], while the proportion of the patients who have a substance use disorder in this study was unknown. Forth, the absence of a comparison group was also another important limitation of our report. Nevertheless, this study represents the first systematic examination of the performance of routine TB diagnostic tools in a psychiatric population. In light of the poor performance of current laboratory methods and potential high TB prevalence among the patients with severe mental illness, our results highlight the current diagnostic dilemma of pulmonary tuberculosis among adults with severe mental illness.

## Conclusions

In conclusion, our findings reveal that the current laboratory examinations based on sputum samples seem not to be suitable for the diagnosis of active TB in the persons with severe mental illness. The products using a non-invasive specimen such as urine deserve further evaluation, which may generate benefit for the early diagnosis of TB in this special population.

## Abbreviations

AFB: Acid-fast bacilli
BMI: Body mass index
CCMD-3: Chinese Classification of Mental Disorders Version 3
CT: Computed tomography
L-J: Lowenstein-Jensen
MTBC: *Mycobacterium tuberculosis* complex
PBS: Phosphate buffer
TB: Tuberculosis

## References

[1] World Health Organization. Global Tuberculosis Report 2014, WHO/HTM/TB/2014.08. Geneva: WHO; 2014.

[2] Pang Y, Zhou Y, Zhao B, Liu G, Jiang G, Xia H, Song Y, Shang Y, Wang S, Zhao YL (2012). Spoligotyping and drug resistance analysis of Mycobacterium tuberculosis strains from national survey in China. PLoS One.

[3] Whiteford HA, Degenhardt L, Rehm J, Baxter AJ, Ferrari AJ, Erskine HE, Charlson FJ, Norman RE, Flaxman AD, Johns N (2013). Global burden of disease attributable to mental and substance use disorders: findings from the Global Burden of Disease Study 2010. Lancet.

[4] Ferrari AJ, Norman RE, Freedman G, Baxter AJ, Pirkis JE, Harris MG, Page A, Carnahan E, Degenhardt L, Vos T (2014). The burden attributable to mental and substance use disorders as risk factors for suicide: findings from the Global Burden of Disease Study 2010. PLoS One.

[5] Prince M, Patel V, Saxena S, Maj M, Maselko J, Phillips MR, Rahman A (2007). No health without mental health. Lancet.

[6] Doherty AM, Kelly J, McDonald C, O’Dywer AM, Keane J, Cooney J (2013). A review of the interplay between tuberculosis and mental health. Gen Hosp Psychiatry.

[7] Cavanaugh JS, Powell K, Renwick OJ, Davis KL, Hilliard A, Benjamin C, Mitruka K (2012). An outbreak of tuberculosis among adults with mental illness. Am J Psychiatry.

[8] Haddad MB, Wilson TW, Ijaz K, Marks SM, Moore M (2005). Tuberculosis and homelessness in the United States, 1994–2003. JAMA.

[9] Oeltmann JE, Kammerer JS, Pevzner ES, Moonan PK (2009). Tuberculosis and substance abuse in the United States, 1997–2006. Arch Intern Med.

[10] McQuistion HL, Colson P, Yankowitz R, Susser E (1997). Tuberculosis infection among people with severe mental illness. Psychiatr Serv.

[11] Lopez AG (1994). Tuberculosis and the severely mentally ill. Am J Psychiatry.

[12] Saez H, Valencia E, Conover S, Susser E (1996). Tuberculosis and HIV among mentally ill men in a New York City shelter. Am J Public Health.

[13] Robson D, Gray R (2007). Serious mental illness and physical health problems: a discussion paper. Int J Nurs Stud.

[14] Psychiatry Branch of the Chinese Medical Association. Chinese Classification of Mental Disorders, 3rd ed (CCMD-3). Shandong Science and Technology Publishing Company; 2001.

[15] Kessler RC, Aguilar-Gaxiola S, Alonso J, Chatterji S, Lee S, Ormel J, Ustun TB, Wang PS (2009). The global burden of mental disorders: an update from the WHO World Mental Health (WMH) surveys. Epidemiol Psichiatr Soc.

[16] Pang Y, Wang Y, Zhao S, Liu J, Zhao Y, Li H (2014). Evaluation of the Xpert MTB/RIF assay in gastric lavage aspirates for diagnosis of smear-negative childhood pulmonary tuberculosis. Pediatr Infect Dis J.

[17] Xia H, Song YY, Zhao B, Kam KM, O’Brien RJ, Zhang ZY, Sohn H, Wang W, Zhao YL (2013). Multicentre evaluation of Ziehl-Neelsen and light-emitting diode fluorescence microscopy in China. Int J Tuberc Lung Dis.

[18] Pang Y, Xia H, Zhang Z, Li J, Dong Y, Li Q, Ou X, Song Y, Wang Y, O’Brien R (2013). Multicenter evaluation of genechip for detection of multidrug-resistant Mycobacterium tuberculosis. J Clin Microbiol.

[19] Wang L, Zhang H, Ruan Y, Chin DP, Xia Y, Cheng S, Chen M, Zhao Y, Jiang S, Du X (2014). Tuberculosis prevalence in China, 1990–2010; a longitudinal analysis of national survey data. Lancet.

[20] Xiang YT, Yu X, Sartorius N, Ungvari GS, Chiu HF (2012). Mental health in China: challenges and progress. Lancet.

[21] Ou X, Xia H, Li Q, Pang Y, Wang S, Zhao B, Song Y, Zhou Y, Zheng Y, Zhang Z (2015). A feasibility study of the Xpert MTB/RIF test at the peripheral level laboratory in China. Int J Infect Dis.

[22] Kusznierz GF, Latini OA, Sequeira MD (2004). Quality assessment of smear microscopy for acid-fast bacilli in the Argentine tuberculosis laboratory network, 1983–2001. Int J Tuberc Lung Dis.

[23] Pedrazzoli D, Abubakar I, Potts H, Hunter PR, Kruijshaar ME, Kon OM, Southern J (2015). Risk factors for the misdiagnosis of tuberculosis in the UK, 2001–2011. Eur Respir J.

[24] Shata AM, Coulter JB, Parry CM, Ching’ani G, Broadhead RL, Hart CA (1996). Sputum induction for the diagnosis of tuberculosis. Arch Dis Child.

[25] Minion J, Leung E, Talbot E, Dheda K, Pai M, Menzies D (2011). Diagnosing tuberculosis with urine lipoarabinomannan: systematic review and meta-analysis. Eur Respir J.

[26] Lawn SD, Kerkhoff AD, Vogt M, Wood R (2012). Diagnostic accuracy of a low-cost, urine antigen, point-of-care screening assay for HIV-associated pulmonary tuberculosis before antiretroviral therapy: a descriptive study. Lancet Infect Dis.

[27] Shah M, Ssengooba W, Armstrong D, Nakiyingi L, Holshouser M, Ellner JJ, Joloba M, Manabe YC, Dorman SE (2014). Comparative performance of urinary lipoarabinomannan assays and Xpert MTB/RIF in HIV-infected individuals. AIDS.

[28] Martens WH (2001). A review of physical and mental health in homeless persons. Public Health Rev.

[29] RachBeisel J, Scott J, Dixon L (1999). Co-occurring severe mental illness and substance use disorders: a review of recent research. Psychiatr Serv.

